# Dietary effects on human gut microbiome diversity

**DOI:** 10.1017/S0007114514004127

**Published:** 2014-12-11

**Authors:** Zhenjiang Xu, Rob Knight

**Affiliations:** 1 Biofrontiers Institute, University of Colorado, Boulder, CO, USA; 2 Howard Hughes Medical Institute, University of Colorado, Boulder, CO, USA

**Keywords:** Microbiome, Diet, Gut

## Abstract

The human gut harbours diverse and abundant microbes, forming a complex ecological system that interacts with host and environmental factors. In this article, we summarise recent advances in microbiome studies across both Western and non-Western populations, either in cross-sectional or longitudinal surveys, and over various age groups, revealing a considerable diversity and variability in the human gut microbiome. Of all the exogenous factors affecting gut microbiome, a long-term diet appears to have the largest effect to date. Recent research on the effects of dietary interventions has shown that the gut microbiome can change dramatically with diet; however, the gut microbiome is generally resilient, and short-term dietary intervention is not typically successful in treating obesity and malnutrition. Understanding the dynamics of the gut microbiome under different conditions will help us diagnose and treat many diseases that are now known to be associated with microbial communities.

It is estimated that human body contains as many as 10^14^ microbial cells^(^ ^1^ ^)^, and our appreciation of their contribution to host physiology, disease and behaviour is increasing rapidly^(^ ^2^ ^–^ ^5^ ^)^. This complex community, collectively known as the microbiota (their genes are known as the microbiome), contains diverse viruses, bacteria, archaea and eukaryotes^(^ ^3^ ^,^ ^6^ ^)^. Recent advances in high-throughput sequencing technology, together with the development of bioinformatics techniques, have sparked a tremendous explosion of culture-independent microbiome studies (i.e. studies that do not rely on culture-based techniques, which usually capture only a small fraction of microbial diversity) providing a profound insight into the role of the microbiota in human health^(^ ^7^ ^,^ ^8^ ^)^. For example, gut microbes train the immune system^(^ ^9^ ^)^, protect against opportunistic pathogens^(^ ^10^ ^)^, harvest nutrients and energy from diet^(^ ^11^ ^)^, and ferment non-digestible carbohydrates^(^ ^12^ ^–^ ^14^ ^)^. The disruption of the normal gut microbiota (dysbiosis) is associated with obesity^(^ ^15^ ^,^ ^16^ ^)^, diabetes^(^ ^17^ ^)^, various inflammatory bowel diseases (IBD)^(^ ^18^ ^,^ ^19^ ^)^ and autoimmune diseases^(^ ^20^ ^,^ ^21^ ^)^. We are beginning to understand the baseline states for a healthy microbiota (or, rather, for the diverse array of healthy microbiota found in different healthy people), and in contrast, what constitutes a bad microbiota, by studying the taxonomy of the constituent organisms (revealing who is there) and their genes (revealing what they are capable of doing). Many factors, either exogenous or endogenous, affect the composition of the gut microbiota. These factors include host genotype^(^ ^22^ ^)^, age^(^ ^2^ ^)^ and sex^(^ ^23^ ^)^. However, of all the environmental factors studied to date, diet has the largest known impact on the gut microbiota. Revealing the complex interactions between these factors and microbiota may ultimately help us modulate our microbiome to diagnose and treat microbiome-associated diseases in a personalised way, restoring a healthy microbial community.

## Variability of the human gut microbiome across populations and over time

The human gut microbiota is seeded during birth and mainly develops over the first 3 years of life^(^ ^24^ ^)^. From birth, neonates are exposed to microbes from a variety of sources, and the initial colonisation of their guts depends on the microbes first encountered. The initial composition of the gut microbiota depends on the mode of delivery: babies delivered vaginally harbour gut microbiota resembling microbial communities found in their mothers' vaginas, whereas those born via Cesarean section apparently acquire microbes from the skin, dominated by taxa such as *Propionibacterium* and *Staphylococcus* ^(^ ^25^ ^)^. The feeding mode also influences the infant gut microbiota. Breast milk contains nutrients, maternal antibodies and also diverse commensal maternal bacteria including bifidobacteria and lactobacilli^(^ ^26^ ^)^. Compared with formula-fed infants, breast-fed infants have lower levels of *Atopobium* and higher levels of *Bifidobacterium* ^(^ ^27^ ^)^. The diversity in the baby's gut is initially low and increases during development. Over the first 3 years of life, the composition of the microbial community becomes more adult-like, and major microbial shifts are associated with key events such as the introduction of solid food^(^ ^24^ ^)^.

Although the adult gut microbiota are relatively stable within a person (in that serial samples from the same person typically resemble each other more closely than do samples from different people) and are typically dominated by members of the Bacteroidetes, Firmicutes and Actinobacteria, they show great interpersonal and intrapersonal variability. For example, fewer than 50 % of bacterial taxa are shared between monozygotic twins at the species level^(^ ^28^ ^)^. We are starting to appreciate this variability to a greater extent because of the numerous studies facilitated by the methods developed in large-scale projects such as the Human Microbiome Project^(^ ^7^ ^,^ ^8^ ^)^ and the Metagenomics of the Human Intestinal Tract (MetaHIT)^(^ ^29^ ^)^. It has been argued that the gut microbiota can be classified into three broad clusters, or ‘enterotypes’, based on the dominant presence of *Bacteroides*, *Prevotella* or *Ruminococcus* genera^(^ ^30^ ^)^, although statistical support for the three-cluster model is weak and has generally not been replicated in later studies. The idea is controversial, especially because the model of discrete clusters has a clear alternative in terms of continuous gradients in the major taxa that are well supported^(^ ^31^ ^–^ ^34^ ^)^. Intriguingly, despite the considerable variability in the composition of the gut microbiota, metagenomic shotgun sequencing demonstrates that these diverse communities share a core set of gene functions in the microbiome^(^ ^35^ ^)^.

The gut microbial community changes with age. In cross-sectional studies, we see an apparently smooth change in the gut microbiota in the first 3 years of life, followed by relatively subtle changes thereafter. Although few studies have specifically examined the gut microbiota in old age, recent studies on elderly population confirm that they harbour distinct gut microbial communities from younger adults^(^ ^36^ ^)^, and the composition of the microbiota in the elderly subjects correlates significantly with measures of frailty, co-morbidity and nutrition^(^ ^37^ ^)^. Lachnospiraceae and microbial genes involved in the production of SCFA were enriched in elderly subjects living in the community compared with those in long-term care. Another study performing functional profiling of the gut microbiota found an increase in proteolytic function and a decrease in saccharolytic function, in addition to the loss of genes for SCFA production associated with ageing^(^ ^38^ ^)^. However, more studies, and especially prospective longitudinal studies, are needed to clarify how diet and the gut microbiota affect the ageing process and how the gut microbiota are in turn affected by aging itself.

Understanding the variability of the microbiota between people and over time is a prerequisite to discriminating disease states from normal perturbations. Caporaso *et al.* ^(^ ^39^ ^)^ densely sampled microbiota from three body sites (tongue, gut and skin) of two healthy subjects for 6 and 15 months. This time series shows the remarkable temporal dynamics of the gut microbiota of individuals. Although the difference between gut samples from an individual is smaller than those from different individuals or different body sites, there seems to be no core set of taxa present at all time points. However, Faith *et al.* ^(^ ^40^ ^)^ found that in faecal samples sequenced much more deeply, on an average 60 % of strains in the gut can persist over a long period up to 5 years when only a few time points are evaluated, suggesting that either the dynamic range of abundance of particular taxa is large (requiring very deep sequencing to recover them) or strains can be lost but recolonise the host. This temporal consistency assumes that other factors that could affect the microbiota, such as diet and disease status, remain relatively constant.

Most studies of the gut microbiota have been focused on Western populations in Europe, the USA and Canada. It is important to expand these studies to non-Western diet populations in order to fully understand the range of variation of the gut microbiota and how gut microbes have co-evolved with humans. For example, the Japanese population has a unique gene coding for the enzyme porphyranase in the gut bacterium *Bacteroides plebeius*, potentially transferred from marine *Bacteroides* spp. that naturally degrade seaweed, to facilitate the digestion of this component of the Japanese, but not, historically, the US diet^(^ ^41^ ^)^. Gut microbiota of children in a rural African village in Burkina Faso showed an increase in Bacteroidetes and a decrease in Firmicutes relative to European children^(^ ^12^ ^)^. This pattern was also found in a broader cross-cultural comparison of the gut microbiota of people living in the Venezuelan Amazon, rural Malawi and the USA, suggesting that carbohydrate-rich diets increase the amount of *Prevotella* ^(^ ^2^ ^)^. The Hadza hunter-gatherers in Tanzania also harbour unique compositions of microbiota, presumably as a result of their foraging lifestyle^(^ ^42^ ^)^. All these studies demonstrate the heterogeneity of gut microbiota across geographically and culturally diverse populations, although to date not enough populations have been studied to resolve which of these differences are due to diet, which to host genetics and which to environmental exposures, all of which differ among these populations.

## Dietary effects on the gut microbiome

The quantitative contributions of host genetics, environmental factors and diet to shaping the gut microbiota remain largely unknown. Significant associations between host genotypes and their gut microbiota composition have been reported in both human and mouse studies (see Spor *et al.* ^(^ ^43^ ^)^ for a comprehensive recent review). The environment also plays an important role in establishing and modifying the gut microbiota. For example, the close physical contact between humans and companion animals facilitates the acquisition and exchange of microbes. A study of skin, oral and gut microbiomes showed that the microbial communities of cohabiting family members resemble each other and that adults share more microbial taxa with their own dogs than they do with others' dogs^(^ ^44^ ^)^. Having a dog, and the resulting exposure to a diverse microbial community, can be beneficial. Pet ownership in the early, but not later, years of life^(^ ^45^ ^)^ is associated with a significant decrease in the risk of allergic disease^(^ ^46^ ^)^.

As discussed earlier, dietary effects on the gut microbiota and health are often confounded by variation in host genotypes and environmental exposures. Nevertheless, accumulating evidence has suggested that long-term diet is a primary driver of the gut microbiota. As an extreme example, two co-evolution studies of mammals and their gut microbiota has found that both gut microbiota composition and functions are adapted to their diet (herbivorous, carnivorous and omnivorous)^(^ ^47^ ^,^ ^48^ ^)^. In another more recent study, the convergently evolved composition of the gut microbiome in ant-eating mammals, despite their phylogenetic diversity, strongly suggests that diet is the major force shaping their microbiota^(^ ^49^ ^)^. Wu *et al.* ^(^ ^31^ ^)^ showed that the overall structure of the human gut microbiota, at least in a Western population, seems to be driven mainly by long-term dietary effects: *Prevotella* was enriched in people with high-fibre and high-carbohydrate diets, whereas *Bacteroides* was associated with a typical ‘Western’ diet high in protein and fat. The studies highlighted in the previous section also suggest that differences between the gut microbial communities of rural populations and Westerners are largely linked to differences in diet^(^ ^2^ ^,^ ^12^ ^,^ ^42^ ^)^.

Fortunately, we can examine dietary effects more directly with techniques that constrain other interacting factors. Studies of the faecal microbiomes of human monozygotic and dizygotic twins have been helpful in identifying the effects of diet on gut microbiota controlling for host genetics^(^ ^50^ ^)^. In addition, gnotobiotic mice (initially germ-free mice colonised with either a defined set of microbial strains or with a donor faecal sample) provides a well-defined, powerful animal model of the human gut ecosystem to elucidate the effects of nutrients on the microbiome, including taxonomic composition, microbial and host gene expression, and, ultimately, host physiological status in response to controlled changes in diet^(^ ^15^ ^,^ ^51^ ^)^. Using the gnotobiotic mouse model, a recent study found that obese or lean phenotypes can be transmitted to germ-free mice by inoculating them with the gut microbiota from human twins discordant for obesity and that the obese phenotype of the mouse transplanted with the obese microbiota can be rescued by the invasion of a particular species of Bacteroidetes from the mouse transplanted with lean microbiota when they are co-housed^(^ ^14^ ^)^. This rescue and invasion is diminished when the mice are fed with a diet high in saturated fats and low in vegetables and fruit, confirming the dependency of the gut microbiota on diet.

Arguably, diet is among the most easily controlled factors that can potentially manipulate the gut microbiota. Studying the resilience of the microbiota and its patterns of change, during and after dietary intervention, could allow the design of effective nutrition therapy. Ley *et al.* ^(^ ^52^ ^)^ have shown that the ratio of Bacteroidetes:Firmicutes increases in the gut microbiota of people consuming either a fat-restricted or a carbohydrate-restricted low-energy diet for a year and that people who lost more weight showed larger changes in the ratios of these taxa. Two other short-term controlled feeding experiments found that the changes in microbiota composition can be rapid (within 24 h), reproducible and can overwhelm interpersonal variability with a sufficiently extreme diet^(^ ^31^ ^,^ ^53^ ^)^. A dietary intervention study of obese or overweight individuals found that the gene richness (the number of gut microbial genes) is increased and the metabolic status is improved in individuals with low gut microbial gene richness, after a 6-week low-energy, high-protein diet followed by another 6-week weight maintenance diet^(^ ^54^ ^)^, but this improvement is very limited for individuals with already high gut microbial gene richness. Dietary interventions in malnourished humans are not always successful, and the variation may be explained by individual differences in the gut microbiota. Studies of Malawian and Bangladeshi subjects with severe acute malnutrition have shown that their gut microbiota are resilient and often regress back to the state before the food intervention^(^ ^50^ ^,^ ^55^ ^)^, suggesting that longer intervention or a repair of the gut microbiota is needed to effectively treat malnutrition in these cases.

## Conclusions

It remains a challenge to identify the key pathogenic microbiota and to establish a causal (rather than associative) relationship between specific microbes or community states and a given physiological or disease phenotype^(^ ^5^ ^)^. Exactly what role does the microbiota play in obesity, diabetes and other diseases? Is the microbiota shift a result of changes in gut environment or a cause? Does microbiota initiate the effect, or mediate it? Can we design therapeutic food interventions or faecal transplantations to restore a healthy microbiota and cure diseases that stem from dysbiosis? These questions are largely unanswered and will require more mechanistic studies of the gut microbiota. Encouragingly, a recent randomised clinical trial of faecal transplantation demonstrated that it is much more effective at curing recurring infections of *Clostridium difficile* ^(^ ^56^ ^)^ than traditional antibiotic treatment.

Vast resources have been invested in studies of mouse and human microbiota such as the Human Microbiome Project^(^ ^7^ ^,^ ^57^ ^,^ ^58^ ^)^ and the MetaHIT project^(^ ^29^ ^,^ ^59^ ^)^. These projects and their associated technologies have led to a greater understanding of the function of the microbiome. Many of the tools developed and the conclusions reached for the human microbiome can be readily transferred to other host-associated studies. Similar to the human gut microbiota, Firmicutes and Bacteroidetes are also the predominant taxa in horses^(^ ^60^ ^)^, guinea pig^(^ ^61^ ^)^, cats and dogs^(^ ^62^ ^)^. Gastrointestinal microbiome dysbiosis is common in canine and feline diseases, such as diarrhoea and IBD, and the alterations in the bacterial communities of dogs and cats with IBD resemble, at least partially, the dysbiosis in human with chronic intestine inflammation^(^ ^63^ ^)^. For example, *Clostridium* clusters XIVa and IV, which produce beneficial SCFA, are depleted in both humans and dogs with IBD^(^ ^64^ ^)^.

The interactions of host genetics, diet and the gut microbiota are thus convoluted and multi-faceted. In many cases, the effect size may be small compared with the huge variability intrinsic in the data. It will require a large number of samples to reveal such small effects. Citizen science projects such as the American Gut Project (http://www.microbio.me/americangut/) launched in 2013 provide opportunities for the general public to participate. The American Gut Project is one of the largest crowd-funded citizen science projects, with over 3600 participants and 3800 samples collected to date. Such large-scale studies with greater statistical power will allow us to examine the subtle contributions of diet and other environmental factors on the gut microbiota from an integrated perspective.
